# Genome Sequences of *Microbacterium foliorum* Phages BouleyBill and Carostasia, isolated in France

**DOI:** 10.17912/micropub.biology.001492

**Published:** 2025-05-21

**Authors:** Christophe Regeard, Florence Constantinesco-Becker, Anne Lopes, Ana A Arteni, Malika Ouldali, Laura Pieri, Monique Auberdiac, Daniel Delaruelle, Kevin Tambosco, Hakima Abes, Clément Almeida-Monge, Félix Benard, Lucie Boucard, Elsa Chaouat, Juliette Charazac, Caroline Comte, Marie Coutard, Téo Denis, Clarisse Deschamps--Martin, Erwin Filloux, Anastasia Gaultier, Madeleine Gautheret, Hafsa Harrat, Océane Hill, Mattéo Jalmain, Cécile Jolivet, Diane Le Tyrant, Sarah Lopez, Cléa Medin, Camille Outtier, Mélissa Roze, Maria Rubio-Espinal, May-Blue Zeni, Ombeline Rossier

**Affiliations:** 1 Institute for Integrative Biology of the Cell (I2BC), CEA, CNRS, Université Paris-Saclay, Gif-sur-Yvette, France; 2 Faculté des Sciences d’Orsay, Université Paris-Saclay, Orsay, France; 3 Ecole Universitaire de Premier Cycle, Université Paris-Saclay, Orsay, France

## Abstract

Bacteriophages BouleyBill and Carostasia, exhibiting siphovirus morphology, were isolated in France. Both infected *Microbacterium foliorum* strain NRRL B-24224. Their 39,215-bp and 40,393-bp genomes were assigned to subclusters EA4 and EA10, respectively, widening for the first time the known geographical distribution of these subclusters to Europe.

**Figure 1. Plaque and particle morphologies for BouleyBill (A and B) and Carostasia (C and D):**
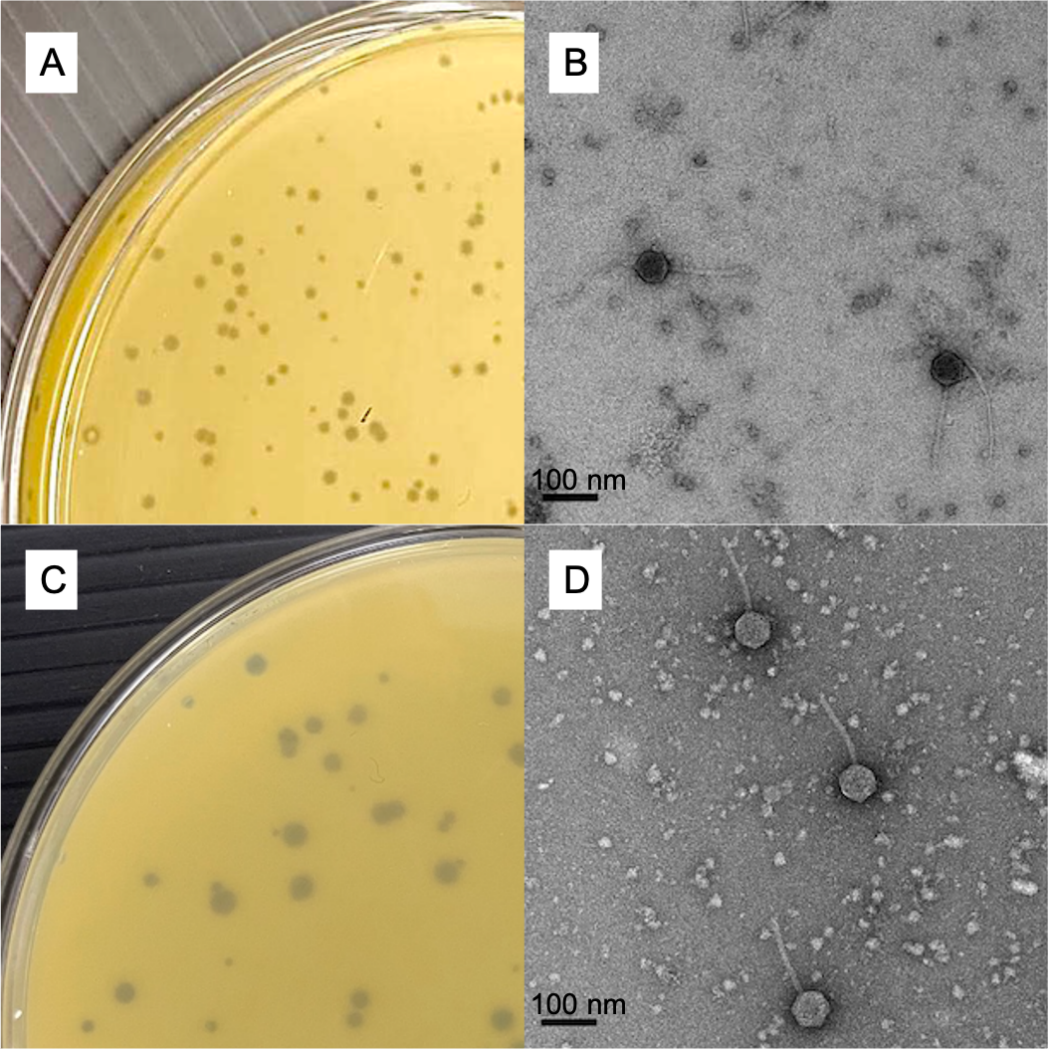
(A and C) Clear plaques observed in the LB agar overlay supplemented with *M* . *foliorum* strain NRRL B-24224 and 1 mM CaCl2 after 24 h at 30°C. (B and D) Negative staining of phages with 2% wt/vol uranyl acetate observed with a Tecnai Spirit microscope operated at 100 kV (TFS) and equipped with a K2 Base 4kx4k direct electron detection camera (Ametek/Gatan). The magnification used was 19,800× with a pixel size of 2.5 Å at the level of the specimen. Scale bar: 100 nm.

## Description

Bacteriophages are abundant and highly diverse viruses that infect bacteria. While approximately 600 different phages infecting the Gram-positive bacterium *Microbacterium foliorum* have been characterized, none have yet been isolated in Europe, leaving their geographic distribution and genetic diversity underexplored (Russell and Hatfull 2017; Jacobs-Sera et al. 2020; Milhaven et al. 2023)(see https://www.phagesdb.org, accessed on 1 December 2024).

Here, we report on the isolation of phages BouleyBill and Carostasia in France (Table 1). Compost or grass sprigs, respectively, were incubated for 1h in LB (5 g/L NaCl; 5 g/L yeast extract; 10 g/L tryptone), shaking at 30°C. Following centrifugation for 15 min at 4000xg, the supernatants were passed through a 0.22-µm filter and plated with top agar (LB 0.75% agar) supplemented with 1 mM CaCl2 and *M* . *foliorum* strain NRRL B-24224 (Russell et al. 2019). BouleyBill and Carostasia formed clear plaques with diameter ranges of 0.5-1 mm and 0.5-2 mm, respectively, after 24h at 30°C. They were further purified through two rounds of single plaque picking and plating (Fig. 1). Negative-staining transmission electron microscopy showed that both phages exhibit siphovirus morphology (Fig. 1). BouleyBill capsids were 55 (±3) nm in diameter and tails were 128 (±7) nm long (n = 13), while Carostasia capsids measured 60 (±1) nm and tails 134 (±5) nm (n = 24).

Phage DNA was extracted from high-titer lysates using the PCI/SDS protocol (https://phagesdb.org/media/workflow/protocols/pdfs/PCI_SDS_DNA_Extraction_2.2013.pdf). A library was prepared using the NEB Ultra II kit and sequenced using an Illumina Miseq instrument (v3 reagents). De novo assembly was performed with Newbler v2.9 as previously described (Russell 2018) and further checked with Consed v.29 (Gordon et al. 1998). The results are listed in Table 1. Based on gene content similarity of at least 35% to phages in the Actinobacteriophage database, phagesDB, both phages were assigned to cluster EA, but to different subclusters (Pope et al. 2017; Russell and Hatfull 2017), i.e. EA4 (containing 11 genomes with BouleyBill) and EA10 (6 genomes including Carostasia).

Gene prediction was performed using DNAmaster v5.23.6 (Pope and Jacobs-Sera 2018), which incorporates Glimmer v3.02 (Delcher et al. 2007) and Genmark v2.5p (Besemer and Borodovsky 2005). Auto-annotation was refined with Aragorn v1.2.41 (Laslett 2004), tRNAscan-SE v2.0 (Chan et al. 2021), Starterator v1.2 (http://phages.wustl.edu/starterator/), and Phamerator v579 (Cresawn et al. 2011). Start sites were selected based on gene length, minimal gaps or overlaps, RBS scores, and BlastP alignment, with Starterator used for further validation. Functional annotation was done with BlastP using NCBI nonredundant or actinobacteriophage databases (e values < 0.001) (Altschul et al. 1990; Russell and Hatfull 2017), and with HHpred (Soding et al. 2005) using databases PDB_mmCIF70_16_Aug, Pfam-A_v37, Uniprot-Swissprot-viral70_3_Nov_2021, and NCBI_Conserved_Domain(CD)_v3.19. Transmembrane domains were predicted using DeepTMHMM v1.0.24 (Hallgren et al. 2022). All software were used with default settings. BouleyBill and Carostasia contained 55 and 63 predicted protein-coding genes, with no putative functional prediction for 50% and 57% of identified genes, respectively.

As previously described for phages belonging to subcluster EA4 (Jacobs-Sera et al. 2020), phage BouleyBill carries a tRNA gene and its tail assembly chaperones are produced through a putative programmed translational -1 frameshift, features that were not identified in Carostasia. BouleyBill contains two genes downstream of the major capsid protein gene, while Carostasia has only one, which differs from the two in BouleyBill. Using Phamerator, a comparison of genomic regions 18,629–21,723 bp in BouleyBill (in particular genes 24, 28 and 29) and 18,678–21,406 bp in Carostasia (genes 23, 26 and 28) shows that the endolysin and two other genes are not conserved. From position 30,564 in BouleyBill and 30,625 in Carostasia to the genome ends, only six genes encode proteins that are homologous between the two phages. Taken together, this report constitutes the first description of *Microbacterium foliorum* phages discovered in Europe. The overall relatedness of BouleyBill and Carostasia with fifteen phages isolated in Northern America or the Caribbean suggests that phages from subclusters EA4 and EA10 have a wide geographical distribution.

Table 1. Isolation, sequencing and genome characteristics of phages BouleyBill and Carostasia

**Table:** 

Phage name	Sample collection site (GPS coordinates)	Sample type	Isolation method	Approx. Shotgun coverage (fold)	No. of 150-bp single-end reads	Genome accession	SRA accession	Genome length (bp)	Genome ends	G + C content (%)	No. of protein-coding genes	No. of tRNAs	Sub-cluster
BouleyBill	Dourdan, FR (48.533211 N, 2.007825 E)	Garden compost	Direct	1,359	375,027	PQ114747	SRX24123901	39,215	Circularly Permuted	64.2	55	1	EA4
Carostasia	Palaiseau, FR (48.713604 N, 2.248526 E)	Sprigs of grass	Direct	1,673	476,899	PQ114742	SRX24123902	40,393	Circularly Permuted	63.8	63	0	EA10
